# Maternal and Cord Blood Lipids in Pregnant Women With Obesity and Their Impact on Neonatal and Placental Biometric Features

**DOI:** 10.1002/osp4.70053

**Published:** 2025-03-12

**Authors:** Fausta Beneventi, Camilla Bellingeri, Irene De Maggio, Carolina Spada, Maria Paola Pandolfi, Alessina Bini Smaghi, Maura Cortese, Elisa Ligari, Claudia Alpini, Arsenio Spinillo

**Affiliations:** ^1^ Dipartimento di Scienze Clinico‐Chirurgiche Diagnostiche e Pediatriche Università di Pavia Pavia Italy; ^2^ Unità Operativa di Ostetricia e Ginecologia 1 Fondazione IRCCS Policlinico San Matteo Pavia Italy; ^3^ Unità Operativa di Laboratorio analisi chimico cliniche Fondazione IRCCS Policlinico San Matteo Pavia Italy

**Keywords:** large for gestational age, obesity, placenta, pregnancy, triglycerides

## Abstract

**Objective:**

Maternal obesity and excessive weight gain during pregnancy predispose to adverse fetal outcomes and health issues for the offspring. Although maternal lipids play an important role in excess fetal fat accretion, previous studies found heterogeneous results regarding which lipid fraction is most involved in excessive fetal growth in maternal obesity and the role of cord lipids. The aim of this study was to evaluate lipid concentrations in maternal and cord blood in pregnant women with and without obesity and to correlate lipid profile with neonatal and placental biometric parameters.

**Methods:**

This is a prospective case‐control study comparing 58 pregnant women with and without obesity enrolled from January 2021 to January 2022 at IRCCS Policlinico San Matteo. Lipid profiles at trimesters and in cord blood were tested. Statistical analysis was conducted with a nonparametric rank‐based approach for longitudinal data analysis.

**Results:**

In both overall and time point analyses, maternal lipid concentrations were higher in participants with obesity than in subjects without obesity. Women with obesity also had higher total cholesterol and triglyceride cord blood concentrations (*p* < 0.001). Among participants with obesity, neonatal and placental weights were positively correlated with triglycerides and the triglycerides/HDL ratio both in maternal and in cord blood. Finally, among subjects with obesity, maternal and cord blood triglycerides and triglycerides/HDL ratio were significantly higher in large for gestational age (LGA) babies compared to non‐LGA (*p* < 0.05).

**Conclusions:**

Compared with controls, obesity in pregnancy is associated with a significant increase in maternal and cord blood lipids, with a positive association between maternal and cord triglycerides and birthweight and placental weight. These findings suggest a further insight into maternal obesity pathophysiology leading to excessive fetal growth, dyslipidemia and insulin resistance in the offspring.

## 1 Introduction

The increasing prevalence of obesity in women of childbearing age [1, 2, 3] and consequently of maternal obesity is a critical cause of short‐ and long‐term problems for the mother as a risk factor for gestational diabetes mellitus (GDM), hypertension, and pre‐eclampsia in pregnancy [4, 5, 6, 7, 8]. Furthermore, maternal obesity and excessive weight gain during pregnancy predispose to adverse fetal outcomes such as preterm birth, macrosomia, impaired fetal growth and neonatal hypoglycemia. In addition, these problems exacerbate obesity and cardiovascular risk in the future for the offspring [4].

Growing evidence suggests that intrauterine factors other than maternal glucose availability contribute to excessive fetal growth in large for gestational age (LGA) infants [9]; indeed, mothers with obesity without diabetes have a notably higher risk of giving birth to babies who are LGA [9, 10, 11]. The impact of GDM on maternal lipids and on fetal growth is widely described in literature [12, 13], on the other hand, evidences of the role of maternal lipid profile in obesity itself in determining fetal weight is limited.

Although maternal lipids may play an important role in excess fetal fat accretion [14], to date, studies on the relationship between maternal lipids and fetal growth have been conducted with different designs, testing different maternal lipids at various gestational age and there is no consensus on which lipid fraction is involved in LGA. Maternal total cholesterol (TC) in previous studies showed a correlation with LGA and small for gestational age (SGA) irrespective of maternal BMI; on the contrary, other reports showed an association of high‐density lipoprotein cholesterol (HDL‐C) more strongly associated with birthweight in women with BMI ≥ 25 [15, 16, 17]. Many data supported maternal triglycerides (TG) as the stronger contributor to excess fetal fat accretion in mothers with obesity [9]. Moreover, the role of cord blood lipids is not completely investigated, neonatal lipoproteins show different structures, compositions, and functionality compared with mothers [18] and evidence on their impact on neonatal weight and childhood adiposity [19, 20] is heterogeneous.

Placentas from women affected by obesity showed changes in lipid metabolism with fewer mitochondria and a lower concentration of acyl carnitines, suggesting a decrease in mitochondrial fatty acids *β*‐oxidation capacity [21]. Nevertheless, recent evidence shows modifications in placental lipid content and metabolic enzyme protein abundance since first trimester irrespectively from obesity [22]. A wider understanding of placental lipid transport and metabolism in uncomplicated and complicated pregnancies could identify maladaptation that occurs in common conditions such as obesity as well as to understand the downstream consequences for the parent–placenta–fetal triad [23].

Previous studies investigating lipid concentrations in pregnancy found an increase in lipid fraction compared to pre‐pregnancy [24, 25, 26] and found higher levels of cholesterol and triglycerides in women with obesity compared with the normal weight category [16]. However, as blood lipids are not routinely measured during pregnancy, there is limited information on normal concentrations during pregnancy and in fetal blood.

Whether the role The aim of this study was to evaluate lipid concentrations in maternal and cord blood in pregnant women affected by obesity compared with controls without obesity and to correlate lipid profile with neonatal and placental biometric parameters, in order to explore the specific role of lipids and placentation as mediators of intrauterine fetal growth in maternal obesity.

## 2 Materials and Methods

This was a prospective case‐control study comparing maternal and umbilical cord lipid serum concentrations between pregnant women with pre‐pregnancy BMI ≥ 30 kg/m^2^ and pregnant women without obesity. All subjects were enrolled from January 2021 to January 2022 among women followed up for prenatal care and delivery at the Department of Obstetrics and Gynecology of Policlinico San Matteo, Pavia, Italy. Inability to give the consent, multiple gestations, pregnancies with chromosomal abnormalities or fetal malformations, primary genetic dyslipidemias, drug/alcohol abuse, pregestational diabetes and GDM in the current pregnancy were exclusion criteria. The Ethical Committee of our Institution approved this study (764‐rcr2013‐bis‐23). We obtained informed written consent from all the women enrolled. The study was conducted according to the Helsinki II declaration. Enrollment took place during the first clinical assessment at 8–10th weeks of pregnancy among consecutive women seen at one of our outpatient prenatal care clinics. Serum samples were collected from all the pregnant women enrolled during the first (8–10th week), the second (18–22th week) and third trimester of pregnancy (28–32th week). All samples were collected at the fasting status. At delivery, we also collected a sample of arterial cord blood. All the serums were sent to the Laboratory of Biochemical‐Clinical Analyses of our hospital and evaluated to measure concentrations of serum fasting TC, HDL‐C, low‐density lipoprotein cholesterol (LDL‐C) and TG concentrations (ADVIA^®^ Chemistry XPT, Siemens Healthcare GmbH, Germany). Triglycerides/HDL (TG/HDL) ratio was also calculated. Maternal blood fasting glucose and glycosylated hemoglobin (HbA1c) evaluated as percentage (%) (ADVIA^®^ Chemistry XPT, Siemens Healthcare GmbH, Germany; coefficient of variation < 2.5%) and insulin (IMMULITE^®^ 2000 Immunoassay System, Siemens Healthcare GmbH, Germany; coefficient of variation 3.5%–7.0%) concentrations were also measured, in order to calculate Homeostasis Model Assessment‐insulin resistance (HOMA‐IR) ([fasting glucose x fasting insulin]/405) [27].

All the women enrolled were followed up and delivered at our Institution; at delivery, placentas were collected and formalin fixed for the evaluation of placental weight and placenta weight percentile [28]. Neonatal weight was evaluated during the neonatologist's clinical examination after birth with a neonatal scale.

A glucose tolerance test (OGTT, 75 g 2 h) at 16–18 and 24–28 weeks of gestation was performed for GDM. According to the International Association of Diabetes and Pregnancy Study Groups Consensus Panel (IADPSG) criteria, diagnosis of GDM was established when one or more abnormal values on fasting plasma glucose and/or on one‐hour and two‐hour plasma glucose of OGTT were found (fasting ≥ 92 mg/dL; 1 h ≥ 180 mg/dL; 2 h ≥ 153 mg/dL) [29]. Excessive gestational weight gain was defined based on Institute of Medicine (IOM) criteria [30] and was calculated from pre‐pregnancy maternal weight. Birth before the 37th week of gestation was defined as preterm delivery. The diagnosis of fetal growth restriction (FGR) and of preeclampsia was made according to international criteria [31, 32]. SGA infants were defined when birth weight was below the 10th percentile and LGA infants were defined if birth weight was over 90th percentile of the Italian population curves [33] adjusting for neonatal sex and gestational age. Data on demographic and clinical characteristics, pregnancy, delivery and neonatal outcomes were collected and stored in a dedicated database.

Categorical variables were compared by Fisher exact test. Kruskal‐Wallis analysis of variance was used to compare continuous variables (non repeated measures) between groups. Spearman rank correlation coefficient was used to test for correlation. To evaluate the effect of trimester of pregnancy (time variable) and of causal factors (treatment variables) on maternal lipid concentrations, we used a nonparametric rank‐based approach for longitudinal data analysis [34] on R (R version 4.3.2. The R foundation for Statistical Computing Vienna Austria 2023). This method is robust to outliers and independent of classical assumptions of linearity and homoscedasticity of parametric linear mixed models. Nonparametric models contained maternal lipids as dependent variables, trimester of pregnancy as time variable and obesity (yes, no) as exposure variables. Hypertension in pregnancy (yes, no) and excessive weight gain in pregnancy (yes, no) were added to the models as covariates. Finally, comparisons of differences in mean concentrations of maternal lipids at each time point were obtained by non‐parametric Friedman balanced analysis of variance and with Tukey‐Kramer's test corrected for multiple comparisons. The sample size of the study was determined using a test for two means in repeated measurements over three times. At the conventional alpha level of 0.05, a sample size of 50 cases and 50 controls offers 95% power to detect a 15% difference in the mean of the variable under investigation between cases and controls (NCSS Pass 2023, Kaysville, Utah, USA).

## 3 Results

During the period of the study, out of a total of 280 consecutive patients seen for antenatal visit at our pregnancy outpatient clinic, 74 women (26.4%) had a pregestational BMI ≥ 30 and 65 (87.8%) gave their informed consent to participate in the study. The subsequent 65 pregnant subjects with a pregestational BMI ≥ 30 kg/m^2^ attending an outpatient clinic after each index case were selected as potential controls. Since the purpose of the study was to evaluate the maternal and fetal lipid profile and the correlation with neonatal biometry, we subsequently excluded from the final analysis pregnant subjects who developed gestational diabetes. In the population with obesity, seven subjects (10.8%) developed gestational diabetes and were excluded from the analysis. Of the 65 enrolled controls, 4 (6.2%) had incomplete data or were lost to follow‐up during pregnancy and three (4.6%) developed gestational diabetes and were excluded from the study. The final groups under study were then composed of 58 healthy controls of normal weight and 58 cases of subjects with BMI ≥ 30.

The main characteristics of the subjects under study and maternal and neonatal outcomes are reported in Table 1. There were no differences in maternal age between the groups studied, whereas women with obesity were more likely to be multiparous, to have a history of previous gestational hypertension, of hypothyroidism, congenital thrombophilia and to develop hypertensive disorder and pre‐eclampsia in current pregnancy after 34 weeks. Compared with controls, women with obesity showed a higher prevalence of excessive weight gain during pregnancy, preterm birth (under 37 weeks), of gestational hypertension, and pre‐eclampsia. Regarding neonatal outcomes, there were no differences in neonatal weight or neonatal weight percentile between the two groups, while among participants with obesity compared to women without obesity, there was a higher prevalence of LGA neonates and placentas had a higher weight percentile.

**TABLE 1:** Maternal clinical features, maternal‐neonatal outcomes and placental macroscopic features.

	Obese (*n* = 58)	Controls (*n* = 58)	Mean difference (95% CI)	*p* value
	Mean (SD)	Mean (SD)		
Age (years)	32.60 (5.29)	32.62 (3.84)	0.017 (−1.68–1.72)	0.98
Pregestational BMI	35.35 (4.19)^a^	22.09 (2.79)	−13.25 (−14.56–−11.94)	< 0.001
Gestational weight gain at term (kg)	9.08 (6.94)	11.91 (3.93)	2.84 (0.76–4.92)	0.008
Gestational week at delivery	38.36 (2.18)^a^	39.38 (1.15)	1.03 (0.38–1.67)	0.002
Neonatal weight	3234.57 (644.65)	3290.95 (343.98)	56.38 (−133.68–246.44)	0.56
Neonatal weight percentile	56.12 (32.52)	47.50 (25.89)	−8.62 (−19.43–2.19)	0.12

	*N* (%)	*N* (%)	Odds ratio (95% CI)
Nulliparous	14 (24.14)^a^	38 (65.51)	0.17 (0.07–0.35)
Previous hypertension	8 (13.79)^a^	0	
Previous diabetes	5 (8.62)	0	
Hypothyroidism	10 (17.24)	4(6.89)	2.81 (0.74–13)
Thrombophilia	4 (6.89)^a^	0	
Preterm delivery (< 37 weeks)	8 (13.8)^a^	0	
Cholestasis	1 (1.72)	0	
Hypertension in pregnancy	16 (27.58)	0	
Excessive weight gain	30 (51.72)^a^	8 (13.79)	6.72 (2.5–19)
Caesarean section	19 (32.75)^a^	9 (15.51)	2.65 (1–7.38)
Operative vaginal delivery	3 (5.17)	5 (8.62)	0.58 (0.09–3.16)
SGA	4 (6.89)	4 (6.89)	1.00 (0.18–5.66)
LGA	17 (29.31)^a^	4 (6.89)	5.58 (1.63–24.3)
FGR	3 (5.17)	0	
NICU admission	5 (8.62)^a^	0	

	Mean (SD)	Mean (SD)	Mean difference (95% CI)	*p* value
Placental weight (g)	514.57 (111.82)	500.22 (89.14)	−14.35 (−51.54–22.85)	0.45
Placental weight percentile	59.26 (31.2)^a^	44.57 (22.5)	−14.69 (−24.69–−4.69)	0.004
Placental diameter (cm)				
Maximum diameter	17.97 (2.35)	18.96 (2.51)	0.99 (0.09–1.89)	0.03
Minimum diameter	14.96 (2.49)	15.84 (2.22)	0.88 (0.01–1.75)	0.04

Abbreviations: FGR, fetal growth restriction; LGA, large for gestational age; NICU, neonatal intensive care unit; SGA, small for gestational age.

^a^ *p* < 0.05 versus controls.

**FIGURE 1:**
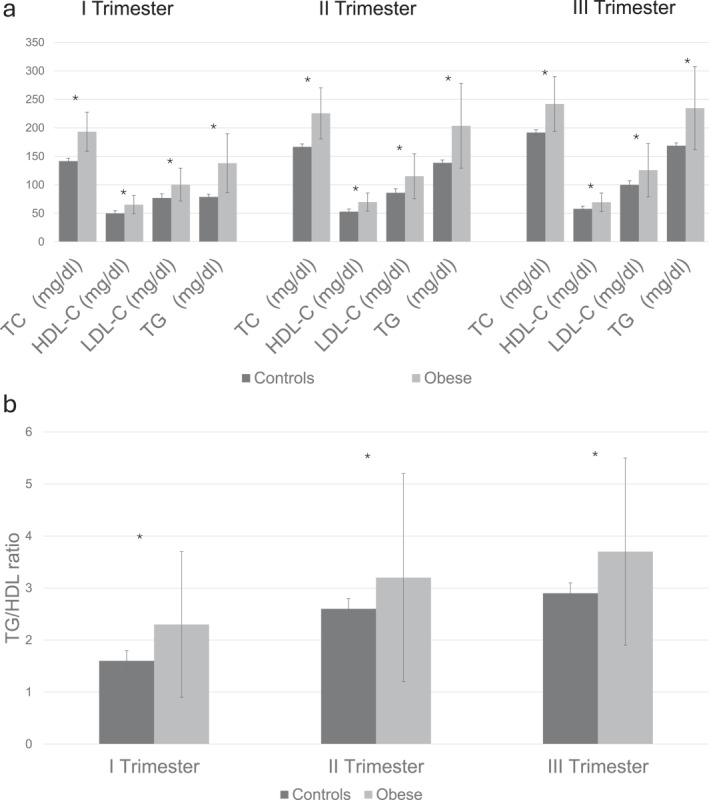
(a) Mean maternal lipid concentrations during pregnancy in obese women compared to controls. (b) Mean TG/HDL ratio during pregnancy trimesters in obese women compared with controls. Error bars represent standard deviation. HDL‐C, High Density Lipoprotein Cholesterol; LDL‐C, Low Density Lipoprotein Cholesterol; TC, Total Cholesterol; TG, Triglycerides. * = *p* < 0.05 compared to controls. Error bars represent standard deviation.

In overall analysis, TC, HDL‐C, LDL‐C, TG, and TG/HDL ratios (all measures expressed in mg/dl) increased significantly during pregnancy both in cases and controls (*p* < 0.01 for all comparisons) (Figure 1a,b). Both in overall and time point analyses, maternal lipids, HOMA‐IR, HbA1c and insulin were all significantly higher among women with obesity than in women without obesity (Table 2). Finally, there were no significant interactions between time and obesity on the maternal concentrations of lipids, HbA1c and insulin. In overall models, excessive weight gain in pregnancy was associated with higher maternal serum concentrations of HbA1c (*p* = 0.002) and of HOMA‐IR (*p* = 0.0006). Compared to controls, in cord blood, women with obesity also had higher total cholesterol (mean difference = 7.9, 95% CI = 3.8–12, *p* < 0.001) and triglycerides (mean difference = 6.8, 95% CI = 3.6–10.1, *p* < 0.001) concentrations and higher TG/HDL ratio (mean difference = 0.37, 95% CI = 0.17–0.56, *p* < 0.001). In overall nonparametric longitudinal analysis, serum concentrations of total cholesterol, HDL, LDL, triglycerides, insulin, HOMA‐IR, HbA1c (%) (*p* < 0.001) and TG/HDL ratio (*p* < 0.05) were higher among obese than in subjects without obesity.

**TABLE 2:** Mean differences in serum maternal lipid and insulin concentrations, HOMA‐IR and HbA1c (%) in women with and without obesity during pregnancy.

	Mean difference I trimester (95% CI)	*p* value^a^	Mean difference II trimester (95% CI)	*p* value^a^	Mean difference III trimester (95% CI)	*p* value^a^
Total cholesterol (mg/dL)	51.46 (35.28–67.64)	< 0.001	58.87 (42.70–75.05)	0.00000	50.29 (34.11–66.47)	< 0.001
HDL (mg/dL)	16.13 (9.92–22.35)	<0.001	16.75 (10.54–22.97)	0.00000	11.41 (5.20–17.62)	< 0.001
LDL (mg/dL)	23.47 (8.68–38.25)	< 0.001	29.14 (14.36–43.92)	0.00000	25.71 (10.93–40.49)	< 0.001
Triglycerides (mg/dL)	59.27 (34.41–84.13)	< 0.001	64.87 (40.02–89.73)	0.00000	65.82 (40.97–90.68)	< 0.001
TG/HDL ratio	0.70 (0.06–1.35)	0.024	0.61 (0.04–1.26)	0.08011	0.75 (0.10–1.41)	0.012
Insulin	9.11 (4.89–13.34)	< 0.001	9.44 (5.22–13.66)	0.00000	12.93 (8.70–17.15)	< 0.001
HOMA‐IR	2.03 (1.02–3.03)	< 0.001	2.12 (1.11–3.12)	0.00000	2.80 (1.79–3.80)	< 0.001
HbA1c (%)	0.62 (0.44–0.80)	< 0.001	0.35 (0.17–0.53)	0.00000	0.26 (0.07–0.44)	< 0.001

^a^ As obtained by non‐parametric Friedman Anova and Tukey‐Kramer's tests corrected for multiple comparisons.

**FIGURE 2:**
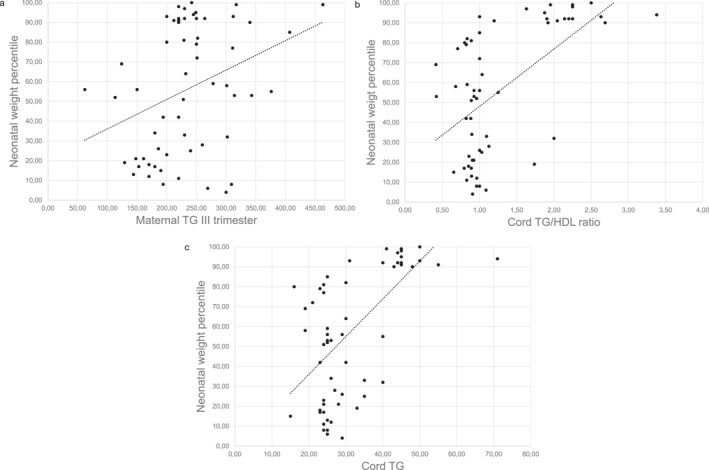
(a–c) Correlations in women with obesity between neonatal weight percentile and (a) Maternal TG. (b) Cord TG. (c) Cord TG/HDL ratio. (a) Spearman Rho = 0.3568, *p* = 0.006. (b) Spearman Rho = 0.5833, *p* = 0.00001. (c) Spearman Rho = 0.5207, *p* = 0.00001. TG, Triglycerides; TG/HDL ratio, Triglycerides/HDL Cholesterol ratio.

We evaluated the correlations of neonatal weight percentile and placental weight percentile according to lipid concentrations in cases and controls. In the control population, no statistically significant correlation was found between lipids and neonatal or placental biometric measures (Supplementary Information S1). On the contrary, in women with obesity, a positive correlation was found between neonatal weight percentile and maternal TG at the third trimester (Spearman Rho = 0.3568, *p* = 0.006), TG in cord blood (Spearman Rho = 0.5833, *p* = 0.00001 vs. controls Spearman Rho = 0.153, *p* = 0.2528) and TG/HDL ratio in cord blood (Spearman Rho = 0.5207, *p* = 0.00001 vs. controls Spearman Rho = −0.07 *p* = 0.59) (Figure 2a–c).

**FIGURE 3:**
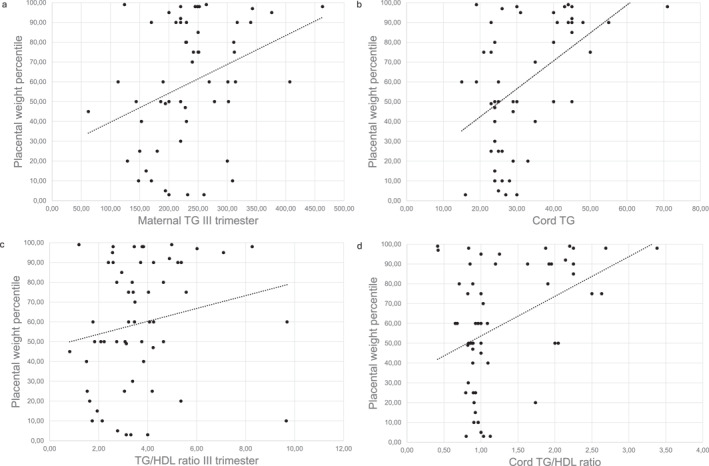
Correlations in women with obesity between placental weight percentile and (a) Maternal TG. (b) Cord TG. (c) Maternal TG/HDL ratio. (d) Cord TG/HDL ratio. (a) Spearman Rho = 0.3605, *p* = 0.0054. (b) Spearman Rho = 0.4135, *p* = 0.0013. (c) Spearman Rho = 0.2758, *p* = 0.036. (d) Spearman Rho = 0.3034 *p* = 0.021. TG, Triglycerides; TG/HDL ratio, Triglycerides/HDL Cholesterol ratio.

Among cases, placental weight percentile correlated positively with TG at the third trimester (Spearman Rho = 0.3605, *p* = 0.0054) and in cord blood (Spearman Rho = 0.4135, *p* = 0.0013), TG/HDL ratio at the third trimester (Spearman Rho = 0.2758, *p* = 0.036) and in cord blood (Spearman Rho = 0.3034 *p* = 0.021) (Figure 3a–d).

Regarding the associations between first trimester maternal lipids and neonatal and placental biometric measures in cases and controls, there were no other significant associations in either group, with the only exception of TG concentrations, which were correlated with neonatal weight percentile (Spearman Rho = 0.2584, *p* = 0.05) and placental weight percentile (Spearman Rho = 0.273, *p* = 0.003) among women with obesity.

As a secondary objective of the study, we evaluated maternal lipids in pregnancies complicated by LGA. Given the low prevalence of LGA in control women (4/58; 6.9%), we restricted the analysis on the association between maternal lipids and LGA only to individuals with BMI ≥ 30 kg/m^2^ (prevalence of LGA = 17/58; 29.3%). In this group, we evaluated the effect of a LGA pregnancy on maternal lipids correcting for nulliparity and excessive weight gain in nonparametric models. There were no differences in maternal TC, HDL‐C, LDL‐C, TG/HDL ratio, HOMA‐IR and insulin during all trimesters between LGA and non‐LGA pregnancies. Among participants with obesity, maternal mean TG in all trimesters was significantly higher in LGA neonates compared with non‐LGA neonates (first trimester 139.95 vs. 101.38 mg/dL; second trimester 195.05 vs. 165.88 mg/dL; third trimester 242.33 vs. 192.64 mg/dL; *p* < 0.05 for all comparisons). However, in the study group with obesity, HDL‐C concentration was lower (mean difference = 5.7, 95% CI = 1.6–9.7, *p* = 0.007) whereas triglycerides (mean difference = −20.3, 95% CI = 15.8–24.7 *p* < 0.001) and TG/HDL ratio (mean difference = 1.17, 95% CI = 0.87–1.47, *p* < 0.001) were higher in LGA as compared to non‐LGA pregnancies.

## 4 Discussion

In our study, we found an increase in blood lipid concentrations from the first to third trimester of pregnancy in both women with and without obesity, with higher levels of TC, LDL‐C, HDL‐C, TG and TG/HDL ratio in women with obesity compared with controls without obesity. Although there are no widely accepted reference ranges for lipid levels in pregnancy, our data are consistent with other reports finding rising serum maternal lipid levels from the first to third trimester [24, 25, 26]. The increase in serum lipids during pregnancy could be attributed to estrogen stimulation and insulin resistance that are hallmarks of metabolic changes during pregnancy. Changes in lipid metabolism, such as fat accumulation, increased tissue lipolysis and maternal hyperlipidemia occur in pregnancy [24].

Moreover, it is known that blood maternal lipid concentrations differ by maternal BMI, as Geraghty et al. found higher levels of cholesterol and triglycerides in mothers with obesity compared with the normal weight category [16]. In obesity, the lipid accumulation in early pregnancy exceeds the storage capacity of adipose tissue, resulting in severe dyslipidemia, hypertriglyceridemia [26] and a higher TG/HDL ratio. In particular, the TG/HDL ratio has been confirmed in the literature as a marker of insulin resistance [35] and of endothelial dysfunction [36] in adults and children, and is correlated with BMI and reduced insulin sensitivity [37, 38]. Regarding the TG/HDL ratio in pregnancy, Wang et al. [39] found an independent association between the TG/HDL ratio at mid‐pregnancy and the risk of GDM and LGA. Moreover, women with a pre‐pregnancy TG/HDL ratio more than 3 showed increased rates of LGA and macrosomia [40].

As expected, compared to controls, the study group with obesity had higher rates of hypertensive disorders, LGA babies and increased neonatal NICU admissions. It is known from literature that elevated lipid levels during pregnancy are associated with adverse pregnancy outcomes [25] and it is suggested that the higher rate of obstetric morbidity could be explained by the atherogenic and proinflammatory effects of excessive lipid concentrations [41].

In cord blood, TC, TG concentrations and TG/HDL ratio were higher among cases compared with controls, with overall lower lipid concentrations in cord blood compared with maternal blood. It is well known that neonatal lipid profile differs in terms of concentrations and distribution of lipoproteins, with lower levels of TC and TG compared with mothers [42]. The likely explanation for this finding is probably the different placental expression of many lipoprotein receptors and transporters [18]. Data on the lipid profile in cord blood in cases of obesity are limited and controversial. In a retrospective cohort of Iranian pregnant women, newborns from women with obesity and with polycystic ovary syndrome (PCOS) showed higher concentrations of TG in cord blood [43]. On the contrary, a small case‐control study reported lower TG in the cord blood in the offspring of mothers with obesity [44], whereas Geraghty et al. [16] found no differences in fetal lipid concentrations between BMI groups. In our cohort, the cord blood TG, TC and TG/HDL ratios were higher in participants with obesity compared with partparticipants without obesity.

In our study, neonatal weight percentile was positively correlated with maternal TG, cord TG, and cord TG/HDL ratio only in the obese study group. The lipid profile in pregnancy has been widely linked to fetal growth in the literature; maternal TG significantly contributes to birthweight, particularly in fetal fat accretion [9]. A meta‐analysis of 42 prospective and retrospective studies (31,402 subjects) found that maternal high TG levels are associated with neonatal birthweight [45] and have significant positive associations with LGA. These associations were strongest in women who had a BMI ≥ 30 prior to pregnancy. Furthermore, when studies that controlled for glucose were excluded, the association between TG concentrations and birthweight remained robust, implying that maternal hypertriglyceridemia may be a risk factor for LGA [9]. In the current study, mothers with prepregnancy BMI ≥ 30 kg/m^2^ and LGA babies had higher triglycerides in all trimesters of pregnancy, indicating that dyslipidemia is a significant risk factor for excessive fetal growth irrespective of diagnosis of diabetes. These findings are consistent with previous research, which found that maternal TG concentrations in the first trimester [25], third trimester [16], or both [46] were associated with neonatal weight and LGA. Moreover, wide studies have highlighted the mediating effect of midpregnancy TG on the relationship between obesity and fetal macrosomia [47, 48].

In the case of obesity, the placenta is exposed to an excess of lipids, which are metabolized and transported to the fetus, promoting fetal hyperinsulinemia and subcutaneous fat accretion [9]. As regards lipid profile in cord blood and its association with birthweight, previous studies found increased TG in cord blood from SGA neonates and increased insulin concentrations in cord blood from LGA neonates [49, 50]. In our population, we found higher levels of TG and increased TG/HDL ratio in LGA babies, which could reflect a specific condition of dyslipidemia and insulin resistance of LGA babies from mothers with obesity and could explain differences with previous studies that were not focused on population affected by obesity.

We also looked at the impact of an excessive gestational weight gain on maternal lipid levels and we found that maternal insulin, TG and HOMA–IR were positively correlated with excessive weight gain during pregnancy, particularly among women with obesity. Excessive weight gain has been linked to fetal complications such as LGA [51], but its relationship with lipid concentrations remains unclear. In a small cohort of women affected by obesity, lipid profile was influenced by pre‐gestational BMI rather than by gestational weight gain [52]. On the other hand, in a larger group of women without obesity, gestational weight gain seemed to modulate the detrimental effects of high maternal TG during pregnancy [53].

In our study, placentas from women with obesity showed a higher weight percentile than women without obesity. This finding is consistent with data from the literature, which confirmed that placentas from women with pregestational obesity were heavier than in controls [54, 55, 56, 57]. Interestingly, in our population with obesity, placental weight percentile was related to third trimester maternal and cord blood TG concentrations. Among determinants of excessive placental weight, higher amounts of nutrients [58] and high concentrations of leptin produced by adipose tissue in mothers affected by obesity [59] could promote proliferation of trophoblasts, which contribute to the increased placental weight [60].

This study highlights the important and specific role of lipids, in particular of maternal triglycerides and TG/HDL ratio in determining neonatal birthweight and placental weight in maternal obesity; moreover, our result arise interest for cord lipids as important in the pathophysiology of excessive fetal growth in LGA neonates from mothers with obesity.

Further studies should focus on the mediator role of the placenta in determining fetal growth in obesity and the effect of maternal dyslipidemia on placental and fetal lipoproteins.

Discovering the specific physiologic path leading from maternal obesity to long‐term impact on metabolic and cardiovascular health in offspring could highlight preventive and therapeutic strategies in pregnancy.

The strengths of this study are the prospective design, the presence of a control population without obesity, the exclusion of GDM, the determination of both maternal (in three trimesters) and cord blood lipids and the correlation with biometric neonatal features.

The rates of a BMI ≥ 30 among pregnant women at their first prenatal visit in Europe range from 9% to 26% without reliable population data from Italy [61]. Among patients attending our prenatal clinic and enrolled in the study, the rate of a BMI ≥ 30 was 26%, signaling the possibility that the single center recruitment could represent the main limitation of the study. However, since this is not a population study, overall relationships between biochemical and clinical variables remain valid.

It is well‐known the effect of physical activity on lipids concentrations [62, 63], though valued with different methods (self‐reported questionnaires, minutes of physical activity, daily count of steps) with interesting evidences also on cord blood lipids [64]. Also maternal diet affected maternal lipid profile, as diet quality was inversely associated with multiple plasma TG, with a potential relation with cardio metabolic health during pregnancy [65]. Following National Italian Guidelines [66] and good clinical practice, subjects in this study were advised follow and a varied and balanced diet was recommended, particularly indicating for obese patients a moderate calorie restriction (20‐25 Kcal/kg) without falling below a caloric intake of 1500 kcal/day and a carbohydrates intake of 175 g/day. A potential limitation of the study could be not considering diet and physical activity as a confounding factors in lipid analysis. However, due to the observational nature of the study, physical activity and diet were not standardized as interventions and a “real life” setting was shown in the study. Nevertheless, life‐interventions such as a high‐quality maternal diet and a daily activity in pregnancy could be potential strategy in order to improve dyslipidemia in pregnancy.

In conclusion, the results of the current study suggest that obesity in pregnancy has a strong correlation with increased maternal and cord blood lipid levels, irrespective of the diagnosis of diabetes. Maternal and cord blood TG and TG/HDL ratio in mothers with obesity showed a significant association with birthweight and LGA, highlighting a possible pathway leading to excessive fetal growth and to fetal dyslipidemia and insulin resistance. Excessive gestational weight gain contributes to the influence of maternal lipid profile in addition to obesity. Maternal and cord blood TG and TG/HDL ratio were correlated with placental weight percentile only in the cohort with obesity. The positive association of TG concentrations with percentile of placental weight suggests a potential association between lipid dysregulation and abnormal placentation. Pregnant women affected by obesity should be accurately follow up during pregnancy with lipid profile evaluation and adequate management of gestational weight gain. Future research should focus on the management of dyslipidemia and obesity in order to reduce obstetric and neonatal risks linked to maternal obesity.

## Author Contributions

F.B., C.B. and I.D.M. conceived the study, acquired, analysed and interpreted data, generated figures, wrote the manuscript; A.S. conceived the study, analysed and interpreted data, proofread the manuscript; C.S., M.P.P., A.B.S., M.C., E.L. and C.A. were involved in literature search, collecting the data and proofread the draft of the manuscript; All authors have contributed to the manuscript substantially and agreed to the final submitted version.

## Conflicts of Interest

The authors declare no conflicts of interest.

## Supporting information

Supporting Information S1
