# Supplementary material for: Maternal and Cord Blood Lipids in Pregnant Women With Obesity and Their Impact on Neonatal and Placental Biometric Features
Source: Obes Sci Pract. 2025 Mar 12;11(2):e70053. doi: 10.1002/osp4.70053 (PMC11900895; doi:10.1002/osp4.70053)
Supplement: Supplementary file 1 — Supporting Information S1 [file OSP4-11-e70053-s001.docx]

## Maternal and cord blood lipids in pregnant women with obesity and their impact on neonatal and placental biometric features

## Fausta Beneventi, Camilla Bellingeri, Irene De Maggio, Carolina Spada, Maria Paola Pandolfi, Alessina Bini Smaghi, Maura Cortese, Claudia Alpini and Arsenio Spinillo

Camilla Bellingeri [camilla.bellingeri@gmail.com](mailto:camilla.bellingero@gmail.com)

Unità Operativa di Ostetricia e Ginecologia 1, Fondazione IRCCS Policlinico San Matteo, Pavia 27100, Italy +39 0382503720

Correlation between Cord TG and birthweight percentile in controls

Spearman's rho = -0.1526 (p= 0.2528)

Correlation between Maternal third trimester TG and birthweight percentile in controls

Spearman's rho = -0.2538 (p=0.0545)

Correlation between cord TG/HDL ratio and birthweight percentile in controls

Spearman's rho = -0.0709 (p=0.5968)

Correlation between third trimester TG/HDL ratio and birthweight percentile in controls

Spearman's rho = 0.1998 (p= 0.1326)

Correlation between third trimester TG and placental weight percentile in controls

Spearman's rho = -0.2914 (p=0.265)

Correlation between third trimester TG/HDL ratio and placental weight percentile in controls

Spearman's rho = 0.0776 (p=0.5628)

Correlation between Cord TG and placental weight percentile in controls

Spearman's rho = -0.0727 (p=0.5878)

Correlation between Cord TG/HDL ratio and placental weight percentile in controls

Spearman's rho = -0.0294 (p=0.8263)
